# Impact of contact lens hygiene risk factors on the prevalence of contact lens-related keratitis in Alexandria-Egypt

**DOI:** 10.1186/s12348-024-00421-1

**Published:** 2024-08-20

**Authors:** Suzan Ibrahim Sakr, Amira Ahmed Nayel, Ahmed Lotfi Khattab, Waad Mahmoud Elhamamsy, Islam Abdelmonaem Abozaid, Ramy Awad, Hager AbdelKhalek Elkazaz, Christeena Saeed Habeel, Raymond Samaha, Alaa Atef Ghaith

**Affiliations:** 1 Cornea Clinic, Alexandria Ophthalmology Hospital, Ministry of Health and Population of Egypt, Alexandria, Egypt; 2 Clinical Pharmacy Department, Alexandria Ophthalmology Hospital, Ministry of Health and Population of Egypt, Alexandria, Egypt; 3 Ophthalmology Department, Gamal Abdelnaser Hospital, Health Insurance, Alexandria, Egypt; 4 Ophthalmology Department, Faculty of Medicine, Alexandria University, Alexandria, Egypt

**Keywords:** Contact lens-related keratitis/risk factors, Contact lens hygiene, Prevalence, Acanthamoeba

## Abstract

**Background:**

This study aimed at measuring the effect of contact lens hygiene risk factors on the prevalence of contact lens-related keratitis and identifying the specific risk factors to both microbial and non-microbial keratitis independently.

**Methods:**

A cross-sectional study was conducted at Alexandria Ophthalmology Hospital from May to October 2023. All contact lens wearers attending the outpatient clinic had undergone face-to-face interviews using a standardized validated questionnaire which included demographic data and contact lenses (CLs) hygiene risk factors. Participants were classified into two groups; normal group and keratitis group. Keratitis group was further subdivided into non-microbial and microbial group.

**Results:**

The study included 245 contact lens wearers; 149 normal cases, 50 (20.4%) contact lens-related non-microbial keratitis (CLNK) cases, and 46 (18.8%) contact lens-related microbial keratitis (CLMK) cases. Sharing contact lenses and eye trauma were significant risk factors for both CLNK (*p*=0.036), (*p*=0.001) and CLMK (*p*=0.003), (*p*=0.017). CLs wear duration for more than 12 hours was associated with an increased risk of CLNK by about 4 times (*p*=0.030) and overnight wear of contact lenses increased the risk of CLNK by 2.6 times (*p*=0.030). Showering or swimming in lenses was identified as a significant risk factor for CLMK (*p*=0.012), moreover washing lenses with tap water increased the risk of CLMK (*p*=0.030).

**Conclusions:**

Poor compliance with contact lenses hygiene rules results in a high prevalence of contact lens-related keratitis. Eye trauma and sharing contact lenses were significant hygiene risk factors for both contact lens-related non-microbial keratitis and contact lens-related microbial keratitis.

**Supplementary Information:**

The online version contains supplementary material available at 10.1186/s12348-024-00421-1.

## Background

Contact lenses are a common means of vision correction that offer cosmetic and optical advantages over spectacles. There are an estimated 140 million individuals who wear contact lenses worldwide [1]. Unhygienic contact lens practices can lead to contact lens-related keratitis, a preventable sight-threatening complication of contact lens wear, which affects thousands of patients and causes a significant load on healthcare services.

Unhygienic CL practices are classified into either modifiable or nonmodifiable risk factors. Modifiable risk factors include extended and overnight contact lens wear, occasional or omission of lens disinfection [2, 3]. poor hand hygiene [4], case hygiene case replacement, and smoking [5]. Nonmodifiable risk factors include sex, young age [6], and socioeconomic status [7]. Systemic risk factors include poor general health, thyroid disease, and diabetes [8]. Lately, lens supply has been implicated as a risk factor where internet or mail order supply of contact lenses have been associated with a higher risk of keratitis compared with obtaining lenses through a contact lens practitioner [9].

Several factors were shown to be correlated to CL related dry eye, including lenses with higher nominal water content, rapid pre-lens tear film thinning time, frequent usage of over-the-counter pain medication, and increased tear film osmolality [10].

For microbial keratitis to develop, at least two conditions always need to be fulfilled. Firstly, there needs to be an alteration or defect in the epithelial cell layer of the cornea (CLNK), and secondly, pathogens need to be present in adequate numbers leading to CLMK. Acanthamoeba keratitis (AK) is considered a serious CLMK caused by Acanthamoeba spp., presented by severe pain and photophobia. Remarkably, Acanthamoeba keratitis has significant importance due to delay in diagnosis and difficulties encountered in its management including long course of treatment and unavailability of commercial eye drops resulting in severe sight-threatening complications [11]. AK is highly related to CL wear since acanthamoeba organisms have been cultured from contact lenses and lens cases [12].

This study aims at measuring the implication of contact lens hygiene on the prevalence of contact lens-related keratitis. There is an underestimation in the literature for certain predisposing risk factors to CLK like eye trauma and sharing lens. This paper discussed CLK specific risk factors for both microbial and non-microbial keratitis independently including those underestimated risk factors to decrease the incidence of contact lens-related keratitis.

## Methods

This was an observational analytical study (A cross-sectional study) including contact lens wearers attending the outpatient clinic of Alexandria Ophthalmology Hospital from May 2023 to October 2023. This study was conducted after approval from the Medical Research Ethics Committee, Ministry of Health and Population, and Alexandria Ophthalmology Hospital approval letter for data collection (9-2023/14).

Participants with a positive history of either refractive or cosmetic contact lenses for the last 3 months before attendance were included. Cases with traumatic ulcers, microbial and non-microbial keratitis with a negative history of contact lens wear were excluded. Participants were given a patient information sheet and consent was obtained and they were able to withdraw from the study at any time.

Cornea team ophthalmologists conducted face-to-face interviews at cornea clinic using a standardized questionnaire “Supplemental file”. The questionnaire was designed to assess the risk factors to CLK, the risk factors included in the questionnaire were based on the literature review. The target population included contact lens wearers (either refractive or cosmetic contact lenses for the last 3 months) of both sexes, aged between 10 and 70 years old, participants were recruited from Alexandria Ophthalmology Hospital. The questionnaire was created and pretested to check content validity by the Alexandria Ophthalmology Hospital cornea team experts to ensure that the questions were properly phrased, clear, concise, and sequenced logically. The questionnaire was available in English and Arabic, the predominant languages of Egypt. A pilot study was conducted and the reliability was checked based on Cronbach’s Alpha. The questionnaire was internally validated by the Alexandria Ophthalmology Hospital Research Committee. Ethical approval was obtained from the Medical Research Ethics Committee, Ministry of Health and Population (9-2023/14). The questionnaire included demographic data about the age, sex, and the purpose of wearing CL (whether refractive or cosmetic contact lenses). Risk factors included overnight use, purchasing CLs from unsafe sources like cosmetics shops or hairdressers, sharing the CLs, showering and swimming with the lenses, and rinsing CLs with tap water. Washing hands prior to insertion and removal of CLs and exposure to an eye trauma while wearing CLs was asked. Duration of wearing CLs was subdivided into wearing them for less than 6 hours,6-12 hours, and more than 12 hours. The frequency of changing CLs solution was classified into changing every use, every week, and every month. Case exchange frequency was divided into less than 3 months, between 3 and 6 months, and more than 6 months.

**Fig. 1:**
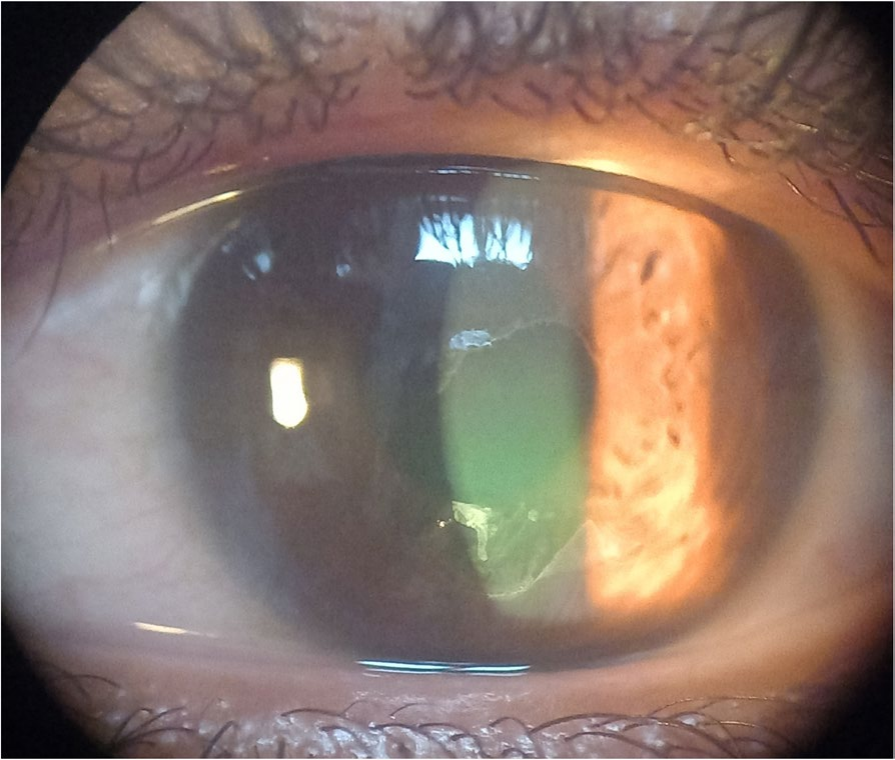
Clinical presentation of non-microbial keratitis (corneal abrasion)

**Fig. 2:**
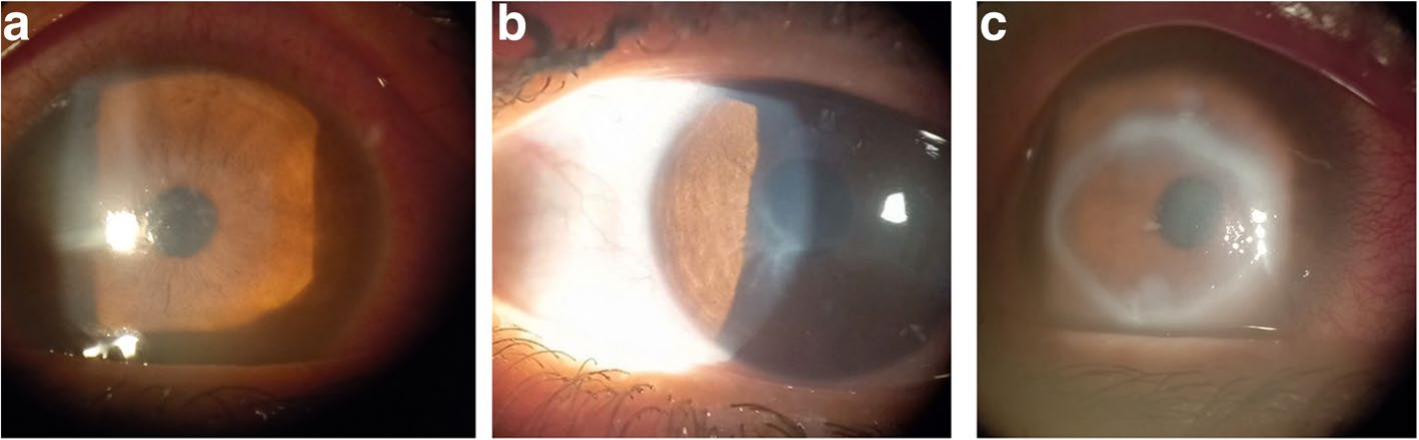
Clinical presentation of contact lens-related Acanthamoeba keratitis; sub-epithelial infiltrates (**a**), perineural infiltrates (**b**), ring infiltrate (**c**)

**Fig. 3:**
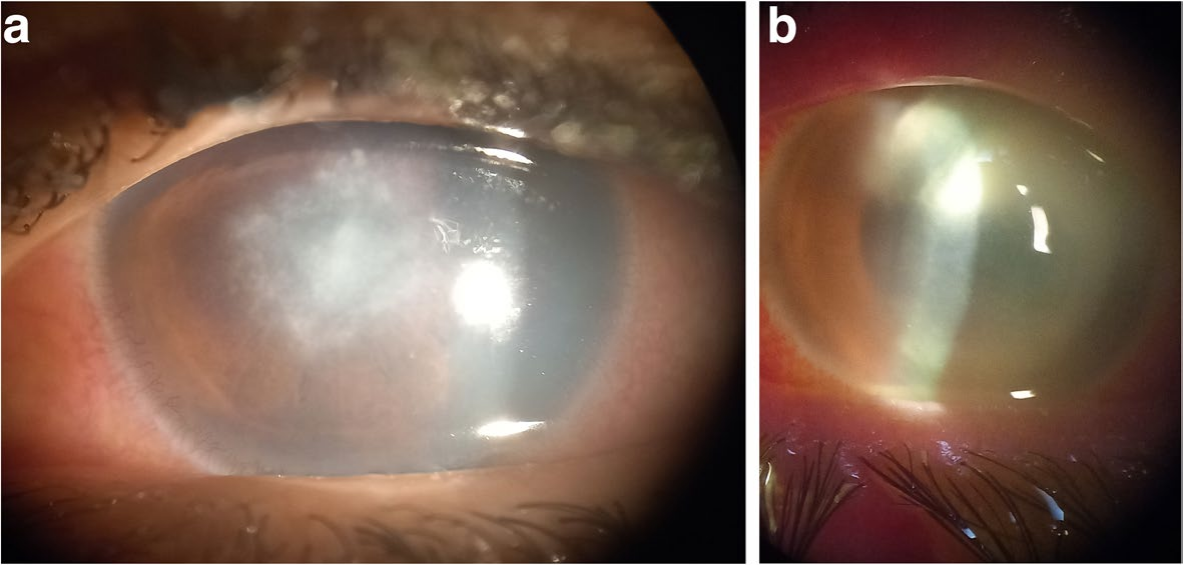
Clinical presentation of bacterial contact lens–associated keratitis; central bacterial stromal infiltration confirmed laboratory as CONS (**a**). Dense serpiginous microbial infiltrates with hypopyon confirmed laboratory as *Streptococcus pneumoniae* (**b**)

All included participants were classified into two groups; normal group and keratitis group. Contact lens-related keratitis was defined as keratitis with a positive history of contact lens wear within the last 3 months prior to examination. Keratitis group was subdivided into non-microbial and microbial groups. Non-microbial keratitis was diagnosed by the presence of an erosion (absence of an epithelial cell layer and intact corneal stroma without infiltration), a corneal ulcer (defects in the epithelium as well as the underlying stroma), or a sterile peripheral corneal infiltrate. Figure 1 shows a slit lamp photo of a case of non-microbial keratitis showing a central epithelial erosion without infiltration. Microbial keratitis was defined as a positive culture from a corneal scrape, a corneal infiltrate (cellular infiltration caused by pathogens, or toxins in the presence of an intact or absent epithelium). Figure 2 shows clinical presentation of different stages of contact lens-related Acanthamoeba keratitis; early sub-epithelial infiltrates(a), radial perineural infiltrates(b), and late ring-shaped infiltrate (c). Figure 3 shows 2 cases of bacterial contact lens–associated keratitis; central dense stromal infiltration confirmed laboratory as CONS (a). Dense serpiginous microbial infiltrates with hypopyon confirmed laboratory as *Streptococcus pneumoniae* (b). All the data from the questionnaire, clinical examination, and laboratory results were collected, and properly analyzed. Univariate and multivariate regression models were used to compare groups.

Contact lens health campaign was held in Alexandria in conjunction with the study from May 2023 to October 2023. The campaign included an Arabic toolkit in the form of posters, flyers, and videos which were posted on social media. The main aim of the campaign was to promote healthy contact lens wear and care for better disease prevention. The campaign organized a contact lens health week in Alexandria Ophthalmology Hospital and trained eye care providers on the guidelines “Supplemental file” [13–15] for better management of contact lens related keratitis.

Data were analyzed using IBM SPSS software package version 20.0. (Armonk, NY: IBM Corp). Categorical data were represented as numbers and percentages. Chi-square test was applied to compare between two groups. Alternatively, Monte Carlo correction test was applied when more than 20% of the cells had an expected count of less than 5. For continuous data, they were tested for normality by the Kolmogorov-Smirnov. Quantitative data were expressed as a range (minimum and maximum), mean, standard deviation and median normally distributed quantitative variables while t-test was used for comparing the two studied groups. Regression to detect the most independent/affecting factor for affecting non-microbial and microbial cases. The significance of the obtained results was judged at the 5% level.

## Results

The study included 245 contact lens wearers; 149 normal cases (60.8%), 50 CLNK cases (20.4.%), and 46 CLMK cases (18.8%). Female gender predominated, with 48 CLNK (96.0%) cases and 44 CLMK (95.7%) cases. Regarding age, the mean age in normal, CLNK and CLMK cases was 27.64, 27.7, and 30.13, respectively. The majority of the studied sample in the three groups used contact lenses for cosmetic purposes (70.5%,66.0%, and 71.7%). On comparing the three studied groups regarding basic demographic data, it was found that there was no significant difference; the three groups were matched regarding age, sex, and purpose of lens use as shown in Table 1.

**Table 1:** Comparison between the three studied groups according to demographic data

	**Normal** **(*n* = 149)**	**Keratitis**		**Test of sig.** **(p**_**1**_**)**	**Test of sig.** **(p**_**2**_**)**
		**Non-microbial** **(*n* = 50)**	**Microbial** **(*n* = 46)**		
**Sex**					
Male	4 (2.7%)	2 (4.0%)	2 (4.3%)	χ^2^= 0.222 (^FE^p_1_ = 0.642)	χ^2^= 0.326 (^FE^p_2_= 0.628)
Female	145 (97.3%)	48 (96.0%)	44 (95.7%)		
**Age (years)**					
Mean ± SD.	27.64 ± 8.66	27.72 ± 9.78	30.13 ± 10.50	t =0.052 (p_1_ = 0.959)	t =1.616 (p_2_ = 0.108)
Median (Min. – Max.)	26.0 (15.0 – 63.0)	26.0 (14.0 – 53.0)	28.0 (14.0 – 61.0)		
**Purpose for lens use**					
Cosmetic	105 (70.5%)	33 (66.0%)	33 (71.7%)	χ^2^= 0.772 (^MC^p_1_=0.695)	χ^2^= 0.440 (^MC^p_2_= 1.000)
Refractive	44 (29.5%)	17 (34.0%)	13 (28.3%)		

*CL* Contact lens *SD* Standard deviation, *t* Student t test, *χ*^*2*^ Chi-square test, *MC* Monte Carlo, *FE* Fisher Exact test, *p*_*1*_ *p*-value for comparing between Normal and Non-microbial, *p*_*2*_ *p*-value for comparing between Normal and microbial

^*^Statistically significant at *p* ≤ 0.05

Table 2 shows the prevalence of CL risk factors among normal and keratitis groups. Sharing contact lenses and eye trauma were significant risk factors in both CLNK (*p*= 0.040), (*p*= 0.001) and CLMK (*p*= 0.004), (*p*= 0.014). Overnight wear of CLs was a significant risk factors in CLNK (*p*= 0.012), while washing CLs with water and showering/swimming during CL wear were significantly associated with CLMK (*p*= 0.028), (*p*= 0.009), respectively. Other risk factors, including CL purchase source, frequency of solution and lens-case exchange, and washing hands before CL use were not statistically significant.

**Table 2:** Comparison between the three studied groups according to CL risk factors

	**Normal** **(*n* = 149)**	**Keratitis**			**Test of sig.** **(p**_**1**_**)**		**Test of sig.** **(p**_**2**_**)**
		**Non-microbial** **(*n* = 50)**	**Microbial** **(*n* = 46)**				
**Contact lens source**							
Cosmetic shops and online	84 (56.4%)	24 (48.0%)	29 (63.0%)		χ^2^= 1.058 (p_1_= 0.304)		χ^2^= 0.641 (p_2_= 0.423)
CL practitioner	65 (43.6%)	26 (52.0%)	17 (37.0%)				
**Washing hands before use**	126 (84.6%)	44 (88.0%)	43 (93.5%)	χ^2^=0.355 (p_1_= 0.551)		χ^2^= 2.417 (p_2_= 0.120)	
**Solution ex-change frequency**							
Every use	90 (60.4%)	25 (50.0%)	23 (50.0%)	χ^2^= 2.099 (p_1_= 0.350)		χ^2^= 1.679 (p_2_= 0.432)	
Every week	31 (20.8%)	15 (30.0%)	13 (28.3%)				
Every month	28 (18.8%)	10 (20.0%)	10 (21.7%)				
**Case exchange frequency (months)**							
<3	78 (52.3%)	23 (46.0%)	19 (41.3%)	χ^2^=0.615 (p_1_= 0.735)		χ^2^=1.772 (p_2_= 0.412)	
3 - 6	36 (24.2%)	14 (28.0%)	13 (28.3%)				
> 6	35 (23.5%)	13 (26.0%)	14 (30.4%)				
**Wear duration (hours)**							
<6	67 (45.0%)	19 (38.0%)	19 (41.3%)	χ^2^=5.052 (p_1_= 0.064)		χ^2^= 2.298 (p_2_= 0.317)	
6 – 12	77 (51.7%)	25 (50.0%)	23 (50.0%)				
>12	5 (3.4%)	6 (12.0%)	4 (8.7%)				
**Overnight wear**	19 (12.8%)	14(28.0%)	7 (15.2%)	χ^2^= 6.293^*^ (p_1_= 0.012^*^)		χ^2^= 0.185 (p_2_= 0.667)	
**Sharing lens**	9 (6%)	8 (16%)	10 (21.7%)	χ^2^= 4.753^*^ (^FE^p_1_= 0.040^*^)		χ^2^=9.850^*^ (^FE^p_2_= 0.004^*^)	
**Washing lens with water**	48 (32.2%)	16 (32.0%)	23 (50.0%)	χ^2^= 0.001 (p_1_= 0.978)		χ^2^= 4.802^*^ (p_2_= 0.028^*^)	
**Shower or swim with CL**	16 (10.7%)	9 (18.0%)	12 (26.1%)	χ^2^= 1.797 (p_1_= 0.180)		χ^2^= 6.734^*^ (p_2_= 0.009^*^)	
**Eye trauma**	17 (11.4%)	16(32.0%)	12 (26.1%)	χ^2^= 11.474^*^ (p_1_= 0.001^*^)		χ^2^=5.981^*^ (p_2_= 0.014^*^)	

*CL* Contact lens *SD* Standard deviation, *t* Student t test, *χ*^*2*^ Chi-square test, *MC* Monte Carlo, *FE* Fisher Exact test, *p*_*1*_ *p*-value for comparing between Normal and Non-microbial, *p*_*2*_ *p*-value for comparing between Normal and microbial

^*^Statistically significant at *p* ≤ 0.05

## Univariate and multivariate analysis

Sharing contact lenses was a significant risk factor for both CLNK (OR, 2.963; 95% CI, 1.076 to 8.159; *p*=0.036), and CLMK (OR, 4.321; 95% CI, 1.634 to 11.423; *p*=0.003). Additionally, eye trauma was a significant risk factor for both CLNK (OR, 3.654;95% CI, 1.675 to 7.970; *p*=0.001), and CLMK (OR, 2.740; 95% CI, 1.196 to 6.282; *p*=0.017). The OR for eye trauma in the multiple regression analysis was 3.468 (95% CI, 1.551 – 7.756; *p*=0.002) in CLNK and it was 2.693(95% CI, 1.112 – 6.519; *p*=0.028) in CLMK as shown in Tables 3 and 4.

**Table 3:** Univariate and multivariate analysis of the risk factors affecting CLNK cases from normal cases

**Non-microbial vs** **normal cases**	**Univariate**		**Multivariate**	
	**p**	**OR (95%C. I)**	**p**	**OR (95% C.I)**
**Sharing lens**	0.036^*^	2.963^*^(1.076 – 8.159)	0.261	1.901 (0.621 – 5.823)
**Eye trauma**	0.001^*^	3.654 (1.675 – 7.970)	0.002^*^	3.468^*^ (1.551 – 7.756)
**Overnight wear**	0.014^*^	2.661^*^(1.216 – 5.821)	0.182	1.839 (0.752 – 4.494)
**Wear duration >12 hours**	0.030^*^	3.927^*^ (1.143 – 13.489)	0.209	2.487 (0.600 – 10.306)

*CLNK* Contact lens-related non-microbial keratitis*, OR* Odd`s ratio, *C.I* Confidence interval

^*^Statistically significant at *p* ≤ 0.05

**Table 4:** Univariate and multivariate analysis of the risk factors affecting CLMK cases from normal cases

**Microbial vs** **normal cases**	**Univariate**		**Multivariate**	
	**p**	**OR (95% C.I)**	**p**	**OR (95% C.I)**
**Sharing lens**	0.003^*^	4.321^*^(1.634 – 11.423)	0.016^*^	3.566^*^ (1.265 – 10.052)
**Eye trauma**	0.017^*^	2.740^*^ (1.196 – 6.282)	0.028^*^	2.693^*^ (1.112 – 6.519)
**Shower or swim with them**	0.012^*^	2.934^*^(1.269 – 6.782)	0.022^*^	2.829^*^ (1.162 – 6.893)
**Washing lens with water**	0.030^*^	2.104^*^(1.074 – 4.122)	0.085	1.871 (0.918 – 3.816)

*CLMK* Contact lens-related microbial keratitis, *OR* Odd`s ratio, *C.I* Confidence interval

^*^Statistically significant at *p* ≤ 0.05

**Fig. 4:**
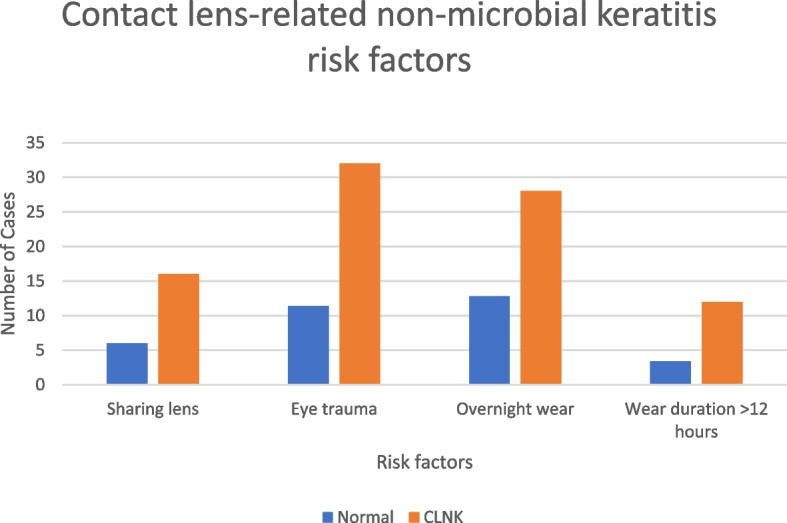
Contact lens-related non-microbial keratitis risk factors

Overnight wear of contact lenses increased the risk of CLNK (OR, 2.661; 95% CI, 1.216 – 5.821; *p*=0.030) and contact lens wear duration for more than 12 hours significantly increased CLNK by 4 times in our analysis (OR, 3.927; 95% CI, 1.143 – 13.489; *p*=0.030). Both were significant in the univariate model but were not significant in the multivariate model. Table 3. Figure 4.

**Fig. 5:**
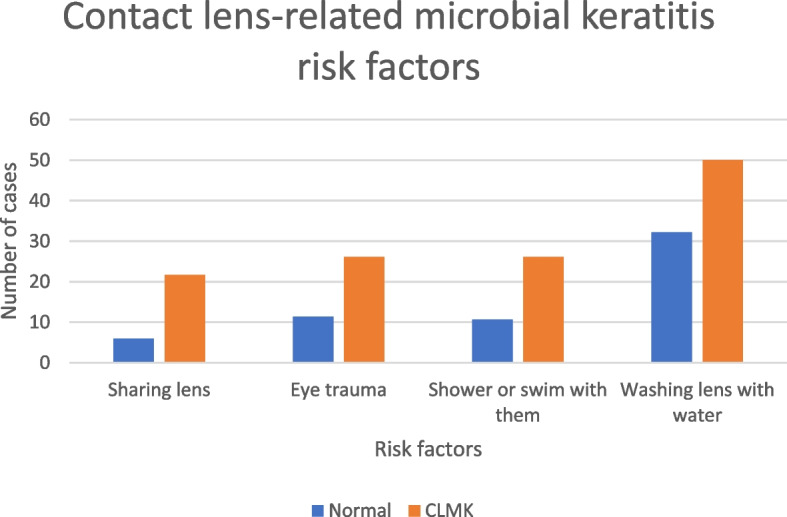
Contact lens-related microbial keratitis risk factors

Showering or swimming while wearing lenses was a significant independent risk factor for CLMK. The univariate regression model showed the OR for showering in lenses compared with normal was 2.934 (95% CI, 1.269 – 6.782; *p*=0.012). The OR for showering in lenses in the multiple regression model was 2.829 (95% CI, 1.162 – 6.893; *p*=0.022). Also Washing lenses with tap water increased the risk of CLMK (OR, 2.104; 95% CI, 1.074 – 4.122; *p*=0.030) in the univariate model but this was not significant in the multivariate model. Table 4. Figure 5.

**Fig. 6:**
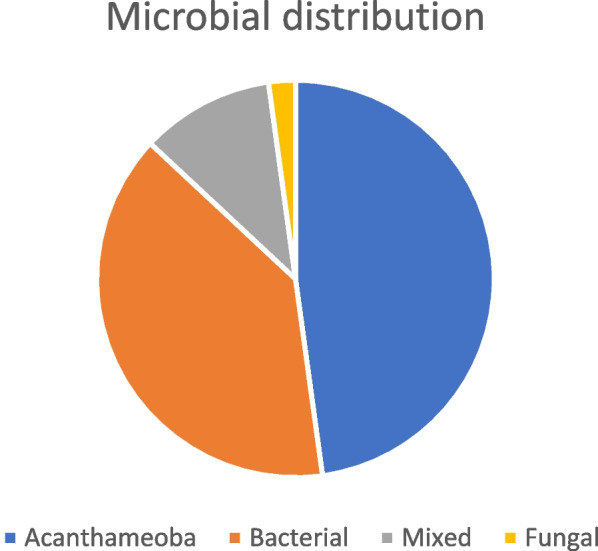
Organism distribution in contact lens-related microbial keratitis

Regarding organism distribution in microbial keratitis, it was found that the most prevalent causative organism to CLMK was Acanthamoeba, which accounts for 22 CLMK cases (47.8%). Bacterial isolates in our study were 18 (39.1%), and Mixed infection was found in 10.9% of cases. Fungi represented only 2.2% of positive scrapes. Table 5. Figure 6.

**Table 5:** Microbial distribution in microbial keratitis

**Microbial distribution**	**(*n* = 46)**
Acanthamoeba	22 (47.8%)
Bacterial	18 (39.1%)
Mixed	5 (10.9%)
Fungal	1 (2.2%)

## Discussion

Contact lens-related keratitis is considered a major problem all over the world and it is responsible for significant visual loss among contact lens users [5, 16]. In our study the prevalence of CLNK and CLMK was 20.4%, and 18.8% which is considered a high percentage compared to studies published by Lam et al, Stapleton et al , and Poggio et al as they estimated the incidence of contact lens-related keratitis at 2 to 20.9 per 10 000 lens wearers per year [4–6]. This is attributed to the nature of the study participants who were seeking medical advice in our hospital. Female gender predominated (96.0% of CLNK cases and 95.7% of CLMK cases). These results were consistent with those reported by Bin Yousef et al (95.8%) [17]. Females are more affected because they use soft contact lenses more than males either for cosmetic or refractive purposes. Regarding age, the mean age in the studied groups was 27 and 30 years, respectively. This was close to the mean age published by Wu et al. (33.8 years) and Dart et al. (32.3) [18, 19]. Oliveira et al. reported younger mean age (23.5 years) since 71% of the participants were university students [20]. The CLAY study results suggest that CL wearers between ages 15 and 25 are at increased risk relative to ages between 8 and 15 years old, this may be attributed to those adolescents and young adult have lower compliance with contact lens hygiene recommendations [21].

Our study analyzed CLK specific risk factors for each of microbial and non-microbial keratitis in contact lens wearers separately including two inadequately studied risk factors; sharing lens and eye trauma. Sharing contact lenses was a risk factor in 16% and 21.7% of CLNK and CLMK cases, which was also reported previously in Saudi Arabia (16.6%) [22]. This percentage was higher than Jordanian university students where sharing CLs accounts only for (1%) [23, 24]. This difference may be attributed to the difference in study participants’ hygiene and culture. Eye trauma before or during wearing contact lenses is another inadequately studied risk factor. An alteration or defect in the epithelial cell layer of the cornea in the presence of a sufficient number of pathogens can lead to CLMK and that was confirmed with our results where coexisting eye trauma was a significant risk factor for both CLNK (*p*=0.001) and CLMK (*p*=0.017).

Interestingly, our study showed that overnight wear of contact lenses increased the risk of CLNK. Similarly previous studies [25, 26], discussed the effect of overnight wear of lenses as a risk factor to CLK. Oxygen diffusion is compromised during sleeping leading to corneal hypoxia. Both extended CL wear and overnight CL wear were associated with the presence of IL-8 and epidermal growth factor both of which lead to CLNK [27]. Regarding CL wear duration for more than 12 hours, we found an increased risk of CLNK in the studied group. These results were not similar to Lubis et al. and Young et al. who reported that CLNK was not correlated with daily lens wear duration, but it was affected by many factors such as contact lens material, lens care solution, eye drops usage, and environment [28, 29]. Moreover, Urgacz et al. reported the occurrence of ulcerative keratitis and superficial punctate keratitis due to the hypersensitivity to preservatives in contact lens care solutions [30]. This is a limitation in our study since we did not investigate contact lens material, lens care solution, and eye drops usage as risk factors to CLK. This point is of importance and needs further research.

In our study, showering or swimming while wearing lenses and washing lenses with tap water significantly increased the risk of CLMK. Acanthamoeba was the most prevalent causative organism to CLMK in our study (47.8%), and this value is higher than that published in the literature by Otri et al (16.9%) [31]. Zhang et al. estimated AK incidence globally, India has the highest incidence at 15.2 cases per million per year, followed by New Zealand, Egypt, Portugal, and the UK (5.2, 5.0, 4.5, and 4.3 cases per million per year, respectively) [32], this variations may be attributed to alterations in sociocultural, environmental, and climate conditions, along with differences in contact lens prescribing practice, eye care availability, and the unawareness of CL wearers of the recommended hygiene procedures, especially regarding water exposure. Acanthamoeba cysts and trophozoites are present in air, dust, soil, and fresh water. They are highly resistant to disinfection with chlorine and are thus not eradicated from tap water [11, 33]. Al-Herrawy et al. isolated Acanthamoeba spp. from finished water samples in Egypt [34]. Moreover, acanthamoeba spp. have been identified in CL or CL cases; variable contamination rates have been verified in different geographic regions; 53.3% in Egypt [15], 15.1% in Korea [35], 10% in Iran [36] and 65.9% in Spain [12]. For this reason, showering or swimming with contact lenses, washing contact lens with tap water all can be considered risk behaviors to acanthamoeba keratitis. Fungi represented only 2.2% of positive scrapes. However, the incidence of fungal keratitis is much higher in India (23–36%) since these microbiological agents are more frequent in tropical and subtropical regions than in temperate regions [37, 38].

Our study showed that 88.0% of CLNK cases and 93.5% of CLMK cases washed their hands before touching lenses, which is similar to that reported from studies in Saudi Arabia (71.9%), and (89.4%) [22, 39]. A lesser percentage was reported from Maldives (44.2%) [40]. This discrepancy may be due to differences between target populations and hygiene practices.

CL purchases by internet order [5] or at unlicensed cosmetics shops [41] were reported to increase the risks of adverse events because these places never provide eye examinations and/or sufficient counseling. Although our results were not consistent with these findings, we emphasize the need to control purchasing CLs from unlicensed stores.

Contact lenses…. are designed for visual and cosmetic purposes, but instead of the visual correction that they were originally invented for …. now accused of causing significant keratitis and visual impairments due to multiple risk factors. CLNK is thought to be one of the predisposing factors to CLMK which is not fully studied and this needs future research.

The strengths of our study are the precise results acquired by face-to-face interviews using a standardized questionnaire and the confirmed diagnosis done clinically by cornea team consultants and laboratory by microbiological investigation.

The findings from this research support the mission of public health ophthalmology which aims to create awareness of adverse events related to CL and to improve CL user behaviors to maintain their health and safety. All CL users should reduce daily wear time by avoiding overnight wearing, and avoiding water exposure. Campaigns are considered a practical solution to contact lens-related keratitis prevention indeed. The study provided updated guidelines to CLMK for healthcare providers for proper management and better outcomes. Eye professionals should follow guidelines regarding CLK diagnosis and management and advise all CL users about regular eye examination and lens hygiene.

## Conclusions

Poor compliance with CL hygiene rules results in the high prevalence of CLK. The greatest personal hygiene risk factor for contact lens-related microbial keratitis and contact lens-related non-microbial keratitis was coexisting eye trauma and sharing contact lenses. Contact lens overnight wear and wear duration for more than 12 hours was associated with an increased risk of CLNK. Showering or swimming while wearing lenses and washing lenses with tap water increased the risk of CLMK. Accordingly, it is absolutely crucial that contact lens wearers practice the necessary care and hygiene in order to avoid serious sequelae to their eyesight.

### Supplementary Information

Supplementary Material 1.

Supplementary Material 2.

## Data Availability

All data relevant to the study are included in the article or uploaded as supplementary information. questionner.

## Abbreviations

CLs: contact lenses
CLNK: contact lens-related non-microbial keratitis
CLMK: contact lens-related microbial keratitis
OR: odds ratio
CI: confidence interval
CLK: contact lens-related keratitis
CONS: coagulase-negative staphylococcus
