# Supplementary material for: Impact of contact lens hygiene risk factors on the prevalence of contact lens-related keratitis in Alexandria-Egypt
Source: J Ophthalmic Inflamm Infect. 2024 Aug 20;14:40. doi: 10.1186/s12348-024-00421-1 (PMC11336145; doi:10.1186/s12348-024-00421-1)
Supplement: Supplementary file 1 — Supplementary Material 1. [file 12348_2024_421_MOESM1_ESM.pdf]

Ministry of Health
Alexandria Health Affairs Directorate
Alexandria Ophthalmology Hospital
Cornea clinic

A Questionnaire for contact lens users

**Date: .... /....../20…**
**Age:**
**Sex: Male/Female**

**From where do you purchase your contact lenses?**		
Hairdressers or beauty shops	Ophthalmologists or contact lens centers	
**The purpose of wearing lenses?**		
To improve vision	Cosmetic	
**Do you wash your hands before wearing them every time?**		
Yes	No	
**How often do you change solution inside the lens case?**		
With each use	Weekly	Monthly
**How often do you change your contact lens case?**		
Less than 3 months	3 to 6 months	More than 6 months
**How many hours do you wear your contact lenses a day?**		
less than 6 hours	6 to 12 hours	More than 12 hours
**Do you wear your contact lenses during sleeping?**		
Yes	No	
**Do you wear your contact lenses during swimming or showering?**		
Yes	No	
**Have you washed the lenses with water?**		
Yes	No	
**Have you borrowed the lenses from any one?**		
Yes	No	
**Have you injured your eye while putting on or removing lenses?**		
Yes	No	

Ministry of Health
Alexandria Health Affairs Directorate
Alexandria Ophthalmology Hospital
Cornea clinic

Cases with positive corneal signs

Patient's name:

Patient's ID:

Clinical presentation:

Normal group	keratitis group	
	**Non-microbial keratitis**	**Microbial keratitis**
		**bacterial**
		**fungal**
		**acanthamoeba**
		**viral**
		**mixed**
