# Supplementary material for: Impact of contact lens hygiene risk factors on the prevalence of contact lens-related keratitis in Alexandria-Egypt
Source: J Ophthalmic Inflamm Infect. 2024 Aug 20;14:40. doi: 10.1186/s12348-024-00421-1 (PMC11336145; doi:10.1186/s12348-024-00421-1)

SYSTEMATIC APPROACH TO CONTACT LENS-RELATED KERATITIS

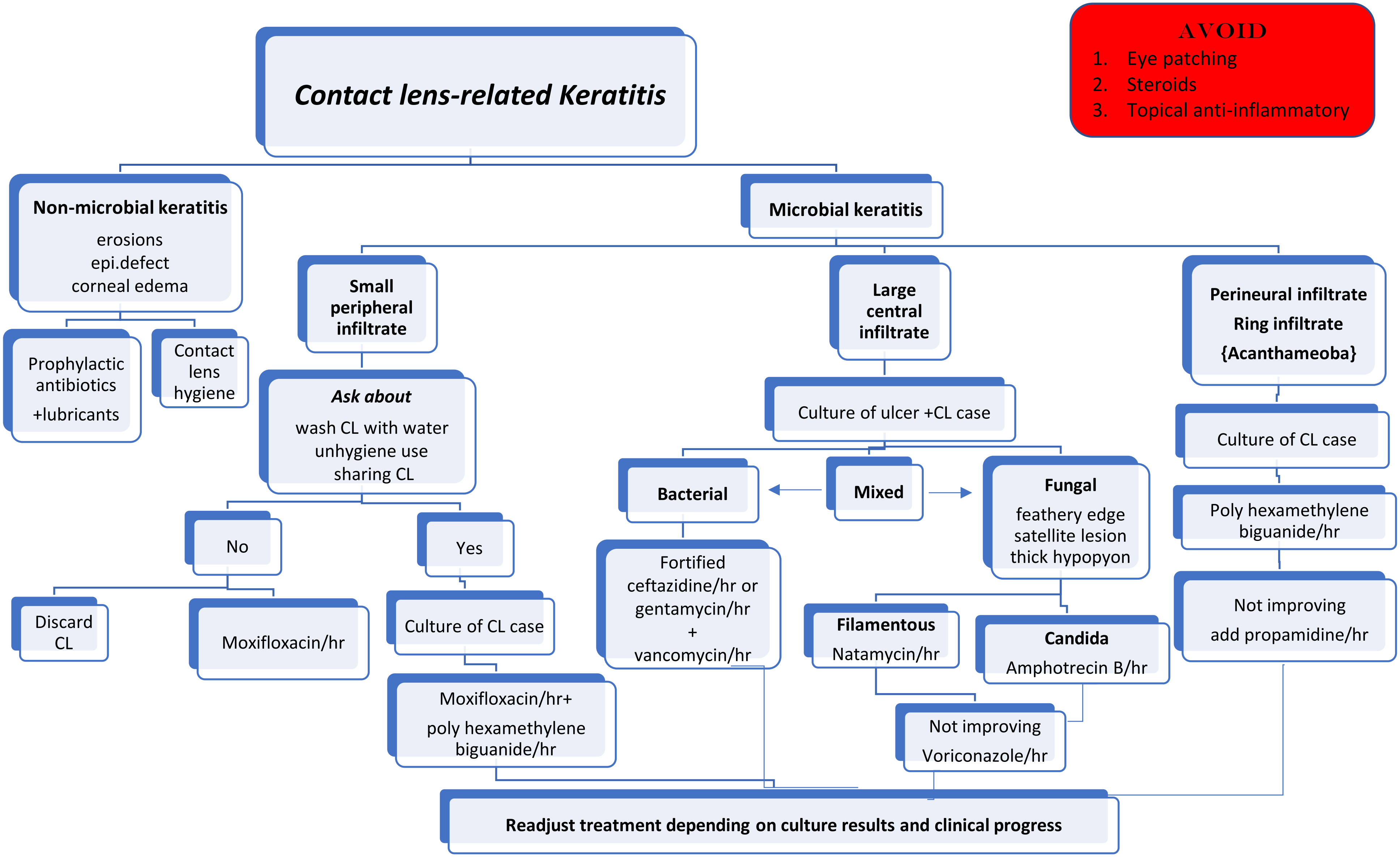

Supplement: Supplementary file 2 — Supplementary Material 2. [file 12348_2024_421_MOESM2_ESM.pdf]
